# Mechanisms Regulating Mitochondrial Transfer in Human Corneal Epithelial Cells

**DOI:** 10.1167/iovs.65.13.10

**Published:** 2024-11-06

**Authors:** Sonali Pal-Ghosh, Beverly A. Karpinski, Himani Datta-Majumdar, Soneha Datta, Shelly Dimri, Jordan Hally, Hugo Wehmeyer, Mary Ann Stepp

**Affiliations:** 1 Department of Anatomy and Cell Biology, GW School of Medicine and Health Sciences, Washington, District of Columbia, United States; 2 Department of Ophthalmology, GW School of Medicine and Health Sciences, Washington, District of Columbia, United States

**Keywords:** cornea, epithelium, neurons, sensory axons, mitochondria

## Abstract

**Purpose:**

The intraepithelial corneal nerves (ICNs) innervating the cornea are essential to corneal epithelial cell homeostasis. Rho-associated kinase (ROCK) inhibitors (RIs) have been reported to play roles in neuron survival after injury and in mitochondrial transfer between corneal epithelial cells. In this study, the mechanisms human corneal limbal epithelial (HCLE) cells use to control intercellular mitochondrial transfer are assessed.

**Methods:**

Mitotracker and AAV1 mitotag eGFPmCherry were used to allow us to study mitochondrial transfer between HCLE cells and neurons in co-cultures and in HCLE cultures. A mitochondrial transfer assay was developed using HCLE cells to quantify the impact of cell stress and inhibition of phagocytosis, gap junctions, and ROCK on mitochondrial transfer, cell adhesion, migration, matrix deposition, and mitochondrial content.

**Results:**

Bidirectional mitochondrial transfer occurs between HCLE cells and neurons. Mitochondrial transfer among HCLE cells is inhibited when gap junction function is reduced and enhanced by acid stress and by inhibition of either phagocytosis or ROCK. Media conditioned by RI-treated cells stimulates cell adhesion and mitochondrial transfer.

**Conclusions:**

Maximal mitochondrial transfer takes place when gap junctions are functional, when ROCK and phagocytosis are inhibited, and when cells are stressed by low pH media. Treatments that reduce mitochondrial content increase HCLE cell mitochondrial transfer. ROCK inhibition in co-cultures causes the release and adhesion of mitochondria to substrates where they can be engulfed by migrating HCLE cells and growing axons and their growth cones.

We have had a long-term interest in how the corneal epithelium supports the corneal sensory nerves that innervate it.^1^ Studies in the mouse have shown that these nerves grow continuously within the corneal epithelium and shed the ends of their nerve terminals in a process regulated diurnally. Terminals are ingested by suprabasal corneal epithelial cells.^2^ The axon terminals contain numerous mitochondria,^3^ and whether corneal epithelial cells degrade all of the axonal mitochondria they ingest or maintain them under homeostasis is not known.

Mitochondrial transfer between cells during cell division is referred to as vertical mitochondrial transfer. Horizontal or intracellular mitochondrial transfer between cells began to be studied intensely two decades ago and is now thought to play numerous roles in cellular and organismal homeostasis and responses to injury.^4^^–^^8^ For example, activated platelets release mitochondria individually and within extracellular vesicles at wound sites.^9^ These mitochondria can circulate in the blood or be transferred to immune cells, vascular endothelial cells, or epithelial cells. In addition, in mouse models, extracellular mitochondria transferred to the injury site after stroke enhance functional recovery.^10^^,^^11^

Although mitochondrial length varies as the organelle undergoes fission and fusion, their diameter is fairly constant ranging between 0.5 µm to 1.0 µm. The mechanisms that regulate the exchange of mitochondria between cells have been widely reported to involve tunneling nanotubes and gap junctions.^7^ Nanotubes range in diameter from 0.05 to 1.5 µm and are capable of shuttling mitochondria between cells. When a nanotube donates mitochondria to another cell, it adheres to and fuses with the recipient cell's plasma membrane. Although data have implicated gap junctions in mitochondrial transfer,^7^^,^^12^ gap junction pores range from 0.8 to 1.6 nm in diameter and are too small to transfer mitochondria between cells. However, gap junctions have also been proposed to stabilize adhesion between the tunneling nanotubes and recipient cells.^13^ Additionally, phagocytosis of mitochondria released into the extracellular space is also thought to play a role in intercellular mitochondrial transfer.^14^

A recent study performed in mice has shown that corneal epithelial cells exchange mitochondria with one another and that a well-characterized RI enhances mitochondrial transfer.^15^ The use of Rho kinase or ROCK inhibitors (RIs) in ophthalmology has increased dramatically over the past decade.^16^^–^^18^ RIs allow corneal endothelial cells maintain their phenotype in vitro and has transformed studies of these cells.^19^ RIs also are neuroprotective in the retina^20^ and are being investigated for use in treating glaucoma.^21^^,^^22^ The widespread effects RIs have on cells are due to the fact that Rho kinases regulate essential signal transduction pathways involving the actin cytoskeleton.^23^ The actin cytoskeleton in turn regulates assembly and disassembly of microtubules and intermediate filaments.

In this in vitro study we demonstrate mitochondrial transfer between human corneal epithelial cells and mouse trigeminal neurons and go on to investigate the cellular mechanisms involved in mitochondrial transfer between human corneal epithelial cells by studying cell stress, and inhibition of phagocytosis, gap junctions, and ROCK using the RI Y-27632. The HCLE cell line we use for these mitochondrial transfer studies was developed in 2003^24^^,^^25^; it is a telomerase immortalized cell line that retains the ability to terminally differentiate in vitro and has been well characterized over the years by us^26^^,^^27^ and others.^28^^–^^30^ The procedures and assays used here benefit from the increased reproducibility obtained using HCLE cells rather than primary human or mouse corneal epithelial cells. The data presented confirm, in corneal epithelial cells, the importance of functional gap junctions and the positive impact inhibition of the ROCK signaling pathway has on mitochondrial transfer.

## Methods

### HCLE Cell Culture

Telomerase immortalized human (hTERT) corneal limbal epithelial (HCLE) cells were generated^31^ and expanded as needed from stocks maintained in liquid nitrogen. Stock vials of HCLE cells were thawed at 37°C and suspended in neutralization medium (500 mL of DMEM/F12 [#11039-021; Gibco, Thermo Fisher Scientific, Waltham, MA, USA], 50 mL calf serum [#A3382001, Gibco, Thermo Fisher Scientific], and 5.5 mL 100X Pen-Strep [#15140-122, Gibco, Thermo Fisher Scientific]). Cells were then resuspended in HCLE media, which consists of supplemented Gibco Keratinocyte SFM (#10724-011, Gibco, Thermo Fisher Scientific); the concentration of BPE used was 25 µg/mL (#13028-014, Gibco, Thermo Fisher Scientific) and of EGF was 0.2 ng/mL (#10450-013, Gibco, Thermo Fisher Scientific) with 5 mL 100x Pen-Strep solution (#15140-148, Gibco, Thermo Fisher Scientific). Cells were grown at 37°C with 5.0% CO_2_ and fed the next day and then every other day until used for experiments. No studies were performed using HCLE cells grown continuously in culture. Every year, a vial from the cell stock is sent for validation, by STR analysis, and for the presence of mycoplasma to the GRC facility at John Hopkins School of Medicine.

### LN332-Coated Glass Coverslips

Fibronectin-Collagen Type 1 (FN/CN) solution was prepared as previously described.^27^ Glass coverslips were placed in 6 well plates and coated with FN/CN for 30 minutes at 37°C. FN/CN was aspirated, and HCLE cells were plated on the coverslips and grown to 100% confluence in HCLE media. LN332 enriched matrix was prepared as described previously.^32^ In brief, freshly prepared 0.02M ammonium hydroxide in 0.1% Triton-x100 was added to the wells with the coverslips for 10 minutes at room temperature. Cellular debris was aspirated, and wells and coverslips were washed two times using 0.1% Triton-x100 buffer followed by two washes with PBS.

### Trigeminal Neuron Cultures and HCLE: Neuron Co-Cultures

From one to three timed pregnant mice, 20 to 24 trigeminal ganglia were dissected from 10 to 12 E11.5 BALB/c mouse embryos; ganglia were pooled in PBS in a microfuge tube kept on ice. After all ganglia were collected, the microfuge tube was spun for one minute at a relative centrifugal force (RCF) of 180*g*, PBS was discarded, and 600 µL Digestion Media was added (10 mL Neurobasal Medium [#21103049, Gibco, Thermo Fisher Scientific], 20 µL 10% BSA [#SH30574.01; Hyclone Laboratories, Logan, UT, USA], and 10 µL Papain [#P3125; Sigma-Aldrich Corp., St. Louis, MO, USA]), and the ganglia were incubated at 37°C for 20 minutes. After incubation, the ganglia were microfuged for one minute at an RCF of 180*g*. Digestion Media was discarded, and the pellet was resuspended and triturated in 600 µL of L15 Complete Medium (L15 Medium [#11415064; Gibco, Thermo Fisher Scientific], 5% FCS [#A3382001; Gibco, Thermo Fisher Scientific], 0.02M HEPES [#15630-080; Gibco, Thermo Fisher Scientific], and 1% Pen-Strep) until dissociated. The tube was then microfuged for three minutes at an RCF of 304*g*, the L15 medium was discarded, and the cells were resuspended in Neurobasal Medium (96 mL Neurobasal Medium [#21103049; Gibco, Thermo Fisher Scientific], 0.63 g Glucose [#A16828.36; Gibco, Thermo Fisher Scientific], 2% B27 serum free supplement [#17504-044; Gibco, Thermo Fisher Scientific], 1% Glutamax supplement [#35050061; Gibco, Thermo Fisher Scientific], 1% Pen-Strep [#15140-148; Gibco, Thermo Fisher Scientific] and 50 µL Human β-NGF [#450-01; PeproTech, Inc., Cranberry, NJ, USA]).

For each assay performed using trigeminal neuron cultures or neuron: HCLE co-cultures, three separate sets of trigeminal ganglion cultures were generated from three different pregnant female mice. For cultures containing neurons only, HCLE LN332-coated coverslips were prepared in six-well plates as described above with one coverslip per well. Sterile cloning cylinders (12 mm top × 13 mm; #F37847-0300; SP BelArt, Wayne, NJ, USA) were placed on the coverslips using a thin layer of sterile silicon grease (#85410; Sigma-Aldrich Corp., St. Louis, MO, USA). 300 µl of Neurobasal medium was placed in the cylinder and, typically, 100 µL of neurons in suspension were plated into the cylinder. Neurons were given one to two hours to adhere before removing media and adding fresh Neurobasal (nb) media, media supplemented with 5 µM RI or with nb HCLE-conditioned media. The optimal concentration of RI used was determined by titration of HCLE cells from 0.625 µM to 10 µM. Because similar results were obtained using 5 µM and 10 µM; the lower concentration of 5 µM was used for these studies. Wells were filled with the same media as within the cylinder so that each well of the six-well plate contained one coverslip with its cloning cylinder. Neurons were incubated at 37°C with 5% CO_2_ for 24 hours. For co-cultures, 100 µl of neurons in suspension were added to subconfluent HCLE cells grown on coverslips in six-well plates. HCLE cells were grown in HCLE media until neurons were to be added; then media was changed to Neurobasal Media prepared as described above.

### Immunofluorescence of HCLE and Trigeminal Ganglion Cells on Coverslips

Immunofluorescence was performed on HCLE cells after fixation with a mixture of 5% glutaraldehyde (#16120; Electron Microscopy Sciences, Hatfield, PA, USA), 2% Formaldehyde (#15712; Electron Microscopy Sciences), in 0.1 M Na Cacodylate buffer (#11654; Electron Microscopy Sciences) for 30 minutes at room temperature (RT). Fixative (concentrate × 2) was added 1:1 to the cell media followed by permeabilization using Triton-x 100 as described previously.^33^ The following antibodies were used: βIII tubulin (1:200; Tuj1; #801201; BioLegend, San Diego, CA, USA) and Tomm 20 (1:200; #PA5-78300; Invitrogen), keratin 14 (1:200; #905301; Biolegend), mCherry (1:200; #MA5-42333; Invitrogen), GFP (1:200; #ab13970; Abcam, Cambridge, MA, USA). F-actin was visualized using phalloidin green 488 (1:500; #A12379; Invitrogen) and red 594 (1:500; #A12381; Invitrogen). Species-specific Alexa-fluor secondary antibodies (488, 594, and 647; Jackson Immunobiologicals, West Grove, PA, USA) were used at 1:500 dilution in blocking buffer. Images were acquired using Nikon Eclipse TS2R (Nikon Inc., Melville, NY, USA) and quantified using NIS Elements BR v5.00 (Nikon Inc.).

### AAV1 Mitotag Transfection

Neurons were generated and plated in cylinders as mentioned above and allowed to adhere for 1-2 hours before adding 3 µL of 7.7 × 10^8^ GC/µL of AAV1 virus (generously provided to us by N. Marsh-Armstrong, UC Davis and the NEI UC Davis core facility) directly to the cylinder overnight. The next day fresh media (with or without HCLE cells depending on the experiment) was added, and the cylinders were removed. Cells were fixed after 24 hours and images were acquired using Nikon Eclipse TS2R with the 100x oil immersion lens.

#### Procedures Used

HCLE cells were used for the procedures described below and were then evaluated using several assays to assess the impact of these treatments on mitochondrial transfer.

##### Acid Stress

To generate media with a pH of 6.5, 100 uL of the freshly prepared 1M HCL solution was added to 10 mL of HCLE media. HCLE cells were washed three times with acidic media and incubated at 37°C in 5% CO_2_ for the times indicated. After incubation in acidic media for one or three hours, acid media was removed, cells were washed three times with normal pH media followed by the assay or allowed to recover for 24 hours and then assayed (1+24h R). Cells were used for the assays described below.

##### Phagocytosis Inhibition

For phagocytosis inhibitor studies, Gö6976 (#2253; Tocris Bioscience, Bristol, UK) was used at a concentration of 10 µM (stock made in DMSO). Appropriate DMSO vehicle dilutions were used for this experiment. Gö6976, referred to as G^0^, and DMSO vehicle were added to the HCLE cells overnight. Control wells with no treatment were also included in each experiment. The following day, cells were washed and used for the assays described below.

##### Gap Junction Inhibition

For Gap Junction inhibitor studies, 18a-glycyrrhetinc (18a-GC) acid (#8503-250 mg; Sigma Aldrich) was used at a concentration of 30 µM (stock made in DMSO). Appropriate DMSO vehicle dilutions were used for the experiment. Gap junction inhibitor and vehicle was added overnight followed by cell migration, phagocytosis, adhesion assay, and mitochondrial transfer studies.

##### RI Studies

Y-27632 dihydrochloride (#1254; Tocris Bioscience) was used at a concentration of 5 µM. The optimal concentration was determined from the literature^34^^,^^35^ and by titration of HCLE cells from 1 µM to 10 µM of RI. Similar results were found using 5 µM and 10 µM; the lower concentration of 5 µM was used for these studies. HCLE cells were grown overnight in media with or without RI. Media was removed, and cells were washed and allowed to grow in HCLE media for assays described below or in Neurobasal media for HCLE: neuron co-culture. Conditioned media was prepared as described in Supplementary Figure S1 from control (CMC) and RI-treated (CMRI) cells. Cells were grown in CMC or CMRI for 24 hours before the assays below were performed.

### Assays

#### CMXROS

CMXROS (#M7512; Invitrogen) was used as per manufacturer's protocol to assess mitochondrial content in cells. A stock solution of 1 mM was made in DMSO and used at a concentration of 100 nM in IHCLE media. CMXROS was added to the unfixed cells for 30 minutes at 37°C, washed twice with PBS, and then fixed for 20 minutes in 4% PFA. Cells were stained with phalloidin to delineate cell boundaries. Six wells were imaged per variable assessed, and four images were acquired per well. For each image acquired, sum intensity and regions of interest were quantified in 25 cells. Therefore *n* = 600 (6 wells × 4 images/well × 25 cells/image) for each variable assessed. Data were expressed as CMXROS SI/ROI (sum intensity/region of interest) with regions of interest defined by phalloidin staining. Each experiment was repeated a minimum of two or three times.

#### Phagocytosis

150 µl of green FluoSphere sulphate beads, 1.0 µm (505/515) beads (#F8852; Invitrogen) was suspended in 300 µL of 1% BSA in PBS and allowed to opsonize serum proteins– for one hour at 37°C on a nutator; beads were sonicated every 15 minutes during opsonization- followed by centrifugation at 4000g for 20 minutes and washed once with PBS. The opsonized and washed beads (150 µL) were resuspended in IHCLE media, and 500 µL of bead suspension was added to each well. Cells were incubated with beads at 37°C for one hour. Unbound beads were then aspirated, and 0.2% trypan blue (#15250-061; Gibco) was added for 10 minutes at RT followed by three washes in PBS. Cells were fixed in 4% PFA for 20 minutes at RT followed by staining with 594 conjugated phalloidin (#A12381; Invitrogen). Six images were acquired at 20× magnification per well. A minimum of three wells were imaged per variable assessed, and each experiment presented was repeated a minimum of three times. Phalloidin 594 delineated cell boundaries (regions of interest or ROI) and data were expressed as CMXROS sum intensity/region of interest or SI/ROI.

#### Cell Adhesion

HCLE cells were grown in 24-well plates to 70% to 80% confluency. After overnight treatment with various stress factors, cells were trypsinized and neutralized, and cell numbers were obtained using the Coulter counter. Equal numbers of control and treated cells were plated out in 24-well plates coated with BSA alone or with FN/CN; nonadherent cells were aspirated 30 minutes after incubation at 37°C. Wells were washed two times with PBS and stained with crystal violet. The dye was extracted with 10% acetic acid, and the ODs measured in a 96-well plate using the Varioskan Lux plate reader and SkanIt RE 7.0.1 (Thermo Scientific) software. Data are expressed as OD representing the adhesion of cells to FN/CN over BSA.

#### Cell Migration

Cells were seeded onto 24-well plates and grown to 70% confluence before imaging on an Olympus IX83 research microscope (Olympus, Tokyo, Japan) equipped with a Proscan motorized stage (Prior Scientific, Inc., Rockland, MA, USA) and placed in a temperature- and CO_2_- controlled chamber (Tokai Hit USA Inc, Bala Cynwyd, PA, USA). Using relief-contrast optics, images (10×) were acquired per well every 10 minutes for 16 hours 40 minutes (100 images). Images were transferred to a workstation equipped with Metamorph image analysis software (Molecular Devices Corporation, San Jose, CA, USA), and velocities of a minimum of 10 cells per well with four to six wells per variable were calculated using the cell track module. For these analyses, the number of cells whose velocities are assessed per variable; *n* was greater than 60 cells per variable. Experiments were repeated a minimum of three times. A visual basic program assisted in data analysis. From each cell tracked; average velocity, net displacement, persistence, and total displacement were calculated; data for velocity were presented.

#### HCLE Matrix Deposition

For studies of matrix deposition, HCLE cells were grown in media with or without RI treatment overnight. Media was removed, and cells were washed and fed with media without RI for 48 hours. For studies involving conditioned media, HCLE cells were grown in CMC or CMRI until confluent; a minimum of two different preparation of CM were used for each assessment. To generate HCLE secreted extracellular matrix, freshly prepared 0.02 M ammonium hydroxide in 0.1% Triton-x100 was added to the cells and left for 10 minutes at RT.^32^ Cellular debris was aspirated, and cells were washed two times using the 0.1% Triton-x100 buffer followed by washing two times with PBS. The matrix deposited by the control and RI treated cells was fixed in 4% PFA fixative. The deposition of ECM was quantified by staining the matrix with an antibody against the γ2 chain of LN332 (1:200; LAMC; #PA521514; Invitrogen). Images were acquired using Nikon Eclipse TS2R, and sum intensity was quantified using NIS Elements BR v5.00. A minimum of six values were obtained for each variable assessed.

#### Mitochondrial Transfer

**Figure 1.**
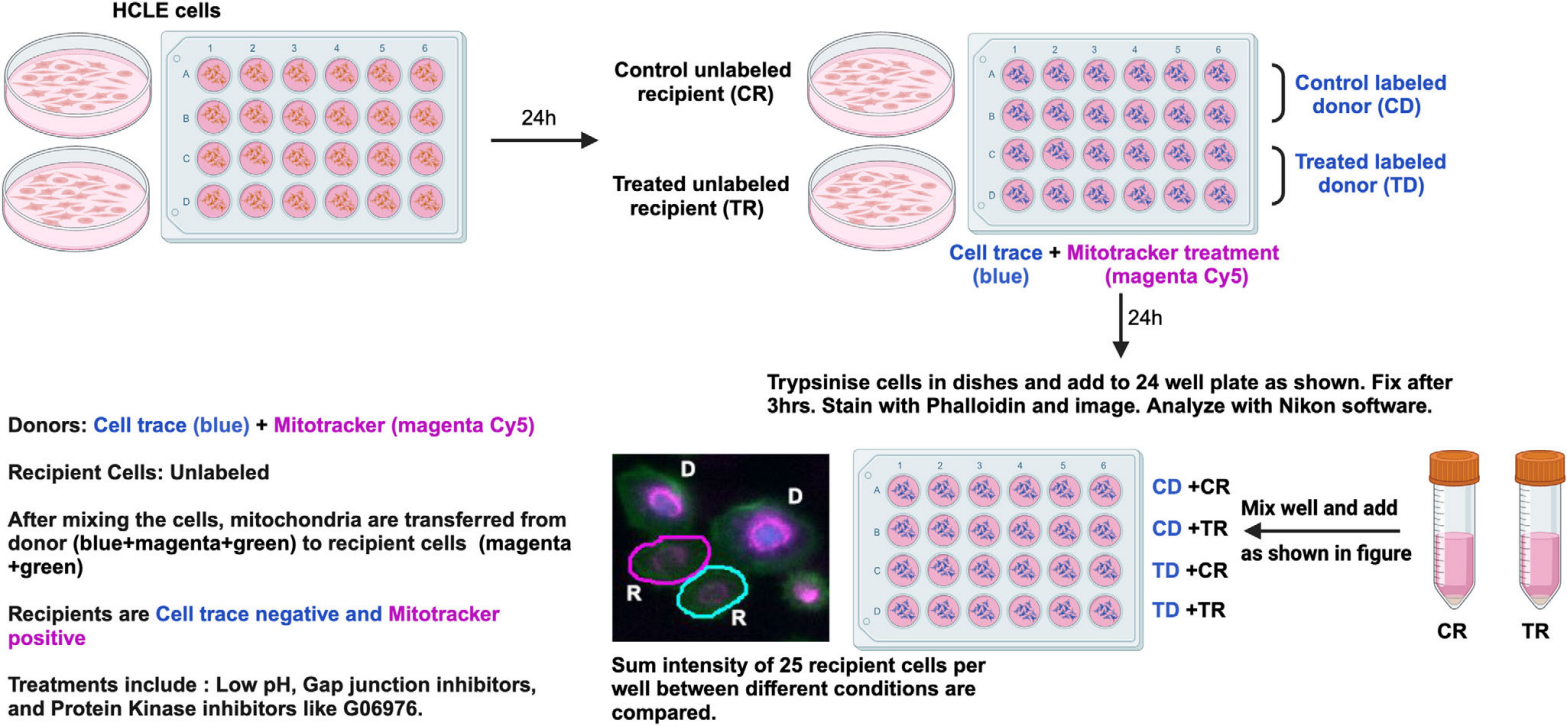
Mitochondrial transfer (mitotransfer) assay. Schematic showing the workflow used to perform mitochondrial transfer assays and quantify results. This approach was used to obtain data on mitochondrial transfer after four treatments of HCLE cells: acid stress, inhibition of phagocytosis, gap junctions, and ROCK inhibitor.

Mitochondrial transfer was quantified as described in the schematic presented in Figure 1. Donor HCLE cells are labeled with Mitotracker Deep Red FM (#M22426; Invitrogen) and CellTrace Violet (#C34557; Invitrogen) as per manufacturer's instructions. Unlabeled recipient HCLE cells are added to wells containing labeled donor cells and mitochondrial transfer assessed after three hours by quantifying the transfer of Mitotracker to recipient cells lacking Cell Trace. Mitochondrial transfer studies were performed on control HCLE cells and cells treated as described above. Cells were stained with phalloidin 488 to delineate cell boundaries (regions of interest or ROI) and data were expressed as sum intensity/region of interest or SI/ROI. Six wells were imaged per variable assessed, and four images were acquired per well. For each image acquired, Mitotracker sum intensity and regions of interest were quantified in 25 cell trace–negative cells per well. Therefore *n* = 600 (6 wells × 4 images/well × 25 cells/image) for each variable.

### Statistics

Data were analyzed for statistical significance using Graphpad Prism. Data for two groups were compared with unpaired *t* test; for three or more groups ANOVA was used when standard deviations between groups were not significantly different. Nonparametric tests (Mann-Whitney to compare two groups and Kruskal-Wallis to compare more than two groups) were used when groups showed significant differences in standard deviations. A single asterisk indicates *P* values between 0.01 to 0.05 and data that are significant, two asterisks indicate *P* values between 0.001 to 0.01 and that data were very significant, and three and four asterisks indicate *P* values between 0.0001 to 0.001 and <0.0001, respectively, and indicate that data were extremely significant. When there are no asterisks provided or the letters ns are seen above a graph, it means the *P* values were greater than or equal to 0.05 and the data were not statistically significant.

## Results

### Mitochondrial Transfer Takes Place Between HCLE Cells and Between HCLE Cells and Trigeminal Neurons

Mitochondria are released by donor cells as components of extracellular vesicles (EVs) or as free mitochondria. In cell culture media, EVs and mitochondria can freely float in solution or become adherent to exosomes and the extracellular matrix deposited by the cells. First, we performed high-resolution imaging of HCLE cells co-cultured with primary mouse E11.5 trigeminal ganglion cells to give us insight into mitochondrial transfer between HCLE cells and trigeminal neurons. For these co-culture studies, HCLE cells and neurons were grown in Neurobasal media prepared as described in the methods section. We realize that this type of interspecies study using mouse and human co-cultures raises concerns, but it was the best option for these studies. Mouse trigeminal neurons are used because we have the experience and the reagents needed to study mouse corneal trigeminal axons in the cornea in vivo. HCLE cells were used because the media required to grow neurons, Neurobasal media, has a higher calcium concentration than optimal for primary mouse or human corneal epithelial cells. The immortalized HCLE cells grew well and did not differentiate when grown in Neurobasal media along with TG neurons.

**Figure 2.**
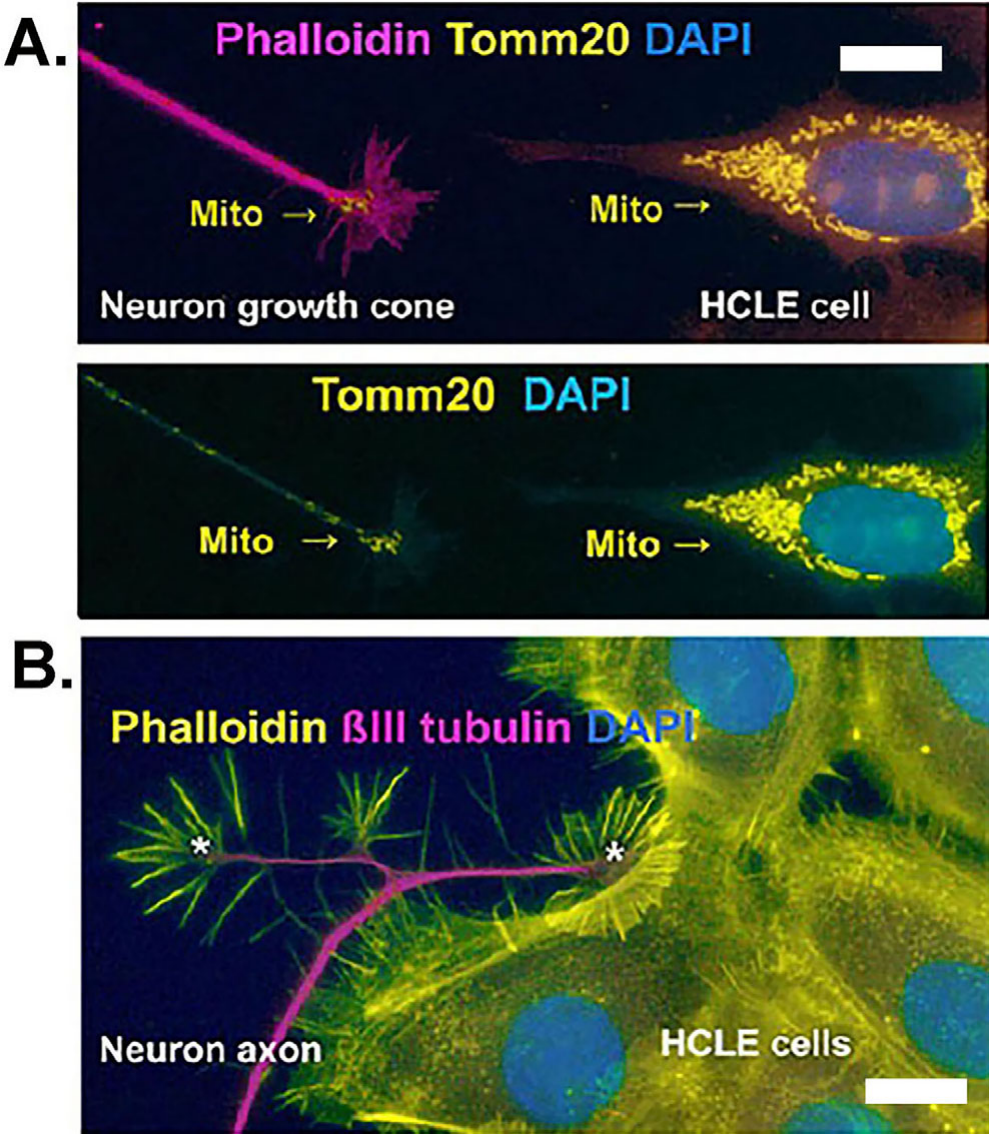
HCLE trigeminal ganglion cell co-cultures show close association between cells and axons. Co-cultures of primary E11.5 mouse trigeminal ganglion neurons and HCLE cells were generated on LN332-coated glass coverslips and fixed. (**A**) Cultures were stained to reveal f-actin using phalloidin (*magenta*) and mitochondria using an antibody against Tomm20 (*yellow*); nuclei are shown in *cyan*. The *top image* shows all three fluorophores to distinguish between axons and HCLE cells whereas the bottom image shows only Tomm20 and DAPI. In HCLE cells, mitochondria localize primarily in perinuclear locations, are elongated, and accumulate within a lamellipodia as it extends toward the axon. Mitochondria are present all along the axon but accumulate at the growth cone. Compared to HCLE cells, the mitochondria in axons are shorter in length. (**B**) Co-cultures were stained to reveal f-actin using phalloidin (*yellow*) and axons using an antibody against βIII tubulin (*magenta*); nuclei are also stained (*cyan*). These images highlight the close association that develops between axons, their axon growth cones, and HCLE cells in co-culture. Phalloidin+ spikes extend from HCLE cells toward axons, and phalloidin+ axon growth cones tunnel underneath HCLE cells at a site where cells are associating with one another in a cluster. *Scale bars:* 5 µm.

Cells were stained with phalloidin (magenta) and an antibody against the mitochondrial protein Tomm20 (yellow) and DAPI (cyan). Figure 2A shows an axon and growth cone of a neuron on the left and an HCLE cell to the right. When the magenta color from f-actin is removed, Tomm20+ mitochondria can be seen in HCLE cells, primarily at perinuclear sites: in the neuron, mitochondria accumulate at the growth cone but can also be seen along the axon where they are being transported to and from the neuron cell body via microtubules. Mitochondria in axons accumulate at growth cones to provide the energy needed for axon elongation and for neurotransmitter release and re-uptake at synapses. Figure 2B shows the close relationship that can develop in these co-cultures between HCLE cells, axons and their growth cones.

**Figure 3.**
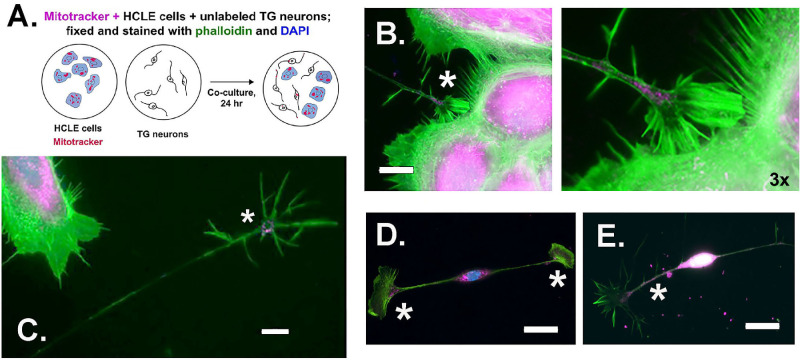
Mitochondrial transfer can be observed from HCLE donor cells and axon recipients using Mitotracker. In the images presented, Mitotracker has been visualized in *magenta*, phallodin in *green*, and DAPI in *blue*. (**A**) The schematic shows in brief how the experiments were performed. (**B**) High-magnification representative image shows Mitotracker + mitochondria in the growth cone of a trigeminal neuron adjacent to an HCLE cell. The image on the right was digitally magnified from the site indicated by the *asterisk*. (**C**) Not all growth cones containing mitochondria derived from HCLE cells are close to the cells as shown in the image. The *asterisk* indicates the growth cone. (**D**, **E**) Examples of neurons projecting short axons from the neuron cell body. Note that the mitochondria derived from HCLE cells localize at the neuron soma, as well as along axons and at growth cones (*asterisks*). The localization of the transferred mitochondria at sites away from the neuron cell body indicates that HCLE-derived mitochondria are not degraded immediately in neuron lysosomes that are located in the soma and axon hillocks. *Scale bars:* 10 µm (**C**, **D**, **E**); 5 µm (**B**).

Mitochondrial transfer studies were next performed using HCLE cells labeled with Mitotracker and Cell Trace as donors and unlabeled mouse primary trigeminal neurons as recipients; to define the cell bodies and axons and their growth cones, phalloidin (green) is also used. TG neurons and HCLE cells were co-cultured for 24 hours. Figure 3 presents a schematic showing how these mitochondrial transfer studies were performed followed by low and high magnification image of HCLE cells labeled with Mitotracker (magenta) surrounding a cluster of neurons (green). These high-resolution images show Mitotracker+ mitochondria transferred from HCLE cells to neurons highlighting mitochondria accumulating in axon growth cones, as well as in neuron cell bodies.

**Figure 4.**
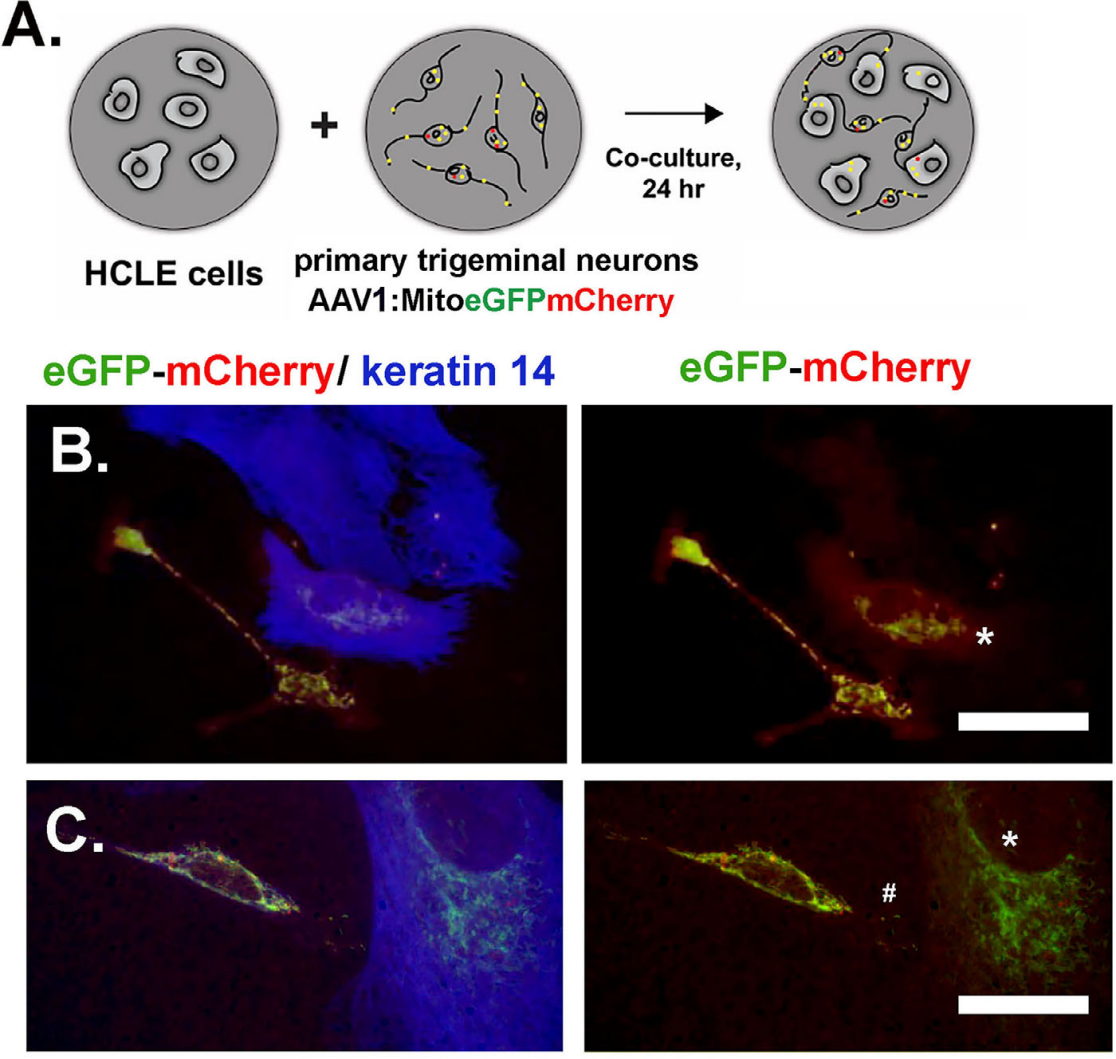
Mitochondrial transfer can be observed between axon donors and HCLE cells by labeling neurons with AAV1-Mito-eGFPmCherry. (**A**) The schematic shows in brief how these experiments were performed. (**B**, **C**) Images of AAV1 Mito-eGFPmCherry transfected neurons co-cultured with HCLE cells. The cultures were fixed, and HCLE cells were visualized using a keratin14 (K14) antibody to distinguish them from neuroprogenitor cells and neurons. The images on the *right* show only eGFPmCherry. Neuronal mitochondria express both eGFPmCherry and are primarily *yellow*. (**B**) The cluster of three HCLE cells shows one cell with numerous *yellow* mitochondria transferred to the HCLE cell from AAV1 transfected neurons (*asterisk*) and another cell with only one or two transferred mitochondria. (**C**) This image shows an eGFPmCherry+ neuron cell body and an HCLE cell to highlight the perinuclear localization of mitochondria transferred to the HCLE cell from eGFPmCherry+ neurons (*asterisk*). These mitochondria express more eGFP than mCherry compared to those shown above. Note the two or three small eGFPmCherry+ mitochondria located between the neuron and the HCLE cell (*pound* *sign*). *Scale bars:* 20 µm (**B**); 10 µm (**C**).

Mitotracker has been found to be toxic to primary trigeminal neurons. To determine whether mitochondrial transfer took place from neurons to HCLE cells, we next used AAV1 Mito eGFP-mCherry^36^ to transfect neurons. Media containing AAV1 was removed, and cells were washed. Twenty-four hours later, we observed labeled mitochondria in neurons. HCLE cells were added, and co-cultures were maintained for 24 hours in Neurobasal media. Figure 4A shows a schematic of how these studies were performed and two representative images (Figs. 4B, 4C) demonstrating the transfer of mitochondria from Mito eGFP-mCherry+ neurons to HCLE cells. In addition to neurons, the primary TG neuron cultures include neuroprogenitor cells that can resemble HCLE cells morphologically. For these studies, cells were fixed, and secondary antibodies against GFP and mCherry were used, and HCLE cells were distinguished from neuroprogenitor cells using an antibody against keratin 14. Of note is that the localization of the transferred mitochondria in the HCLE cells shown in Figures 4B and 4C is perinuclear and similar to that shown for HCLE mitochondria in Figure 2.

**Figure 5.**
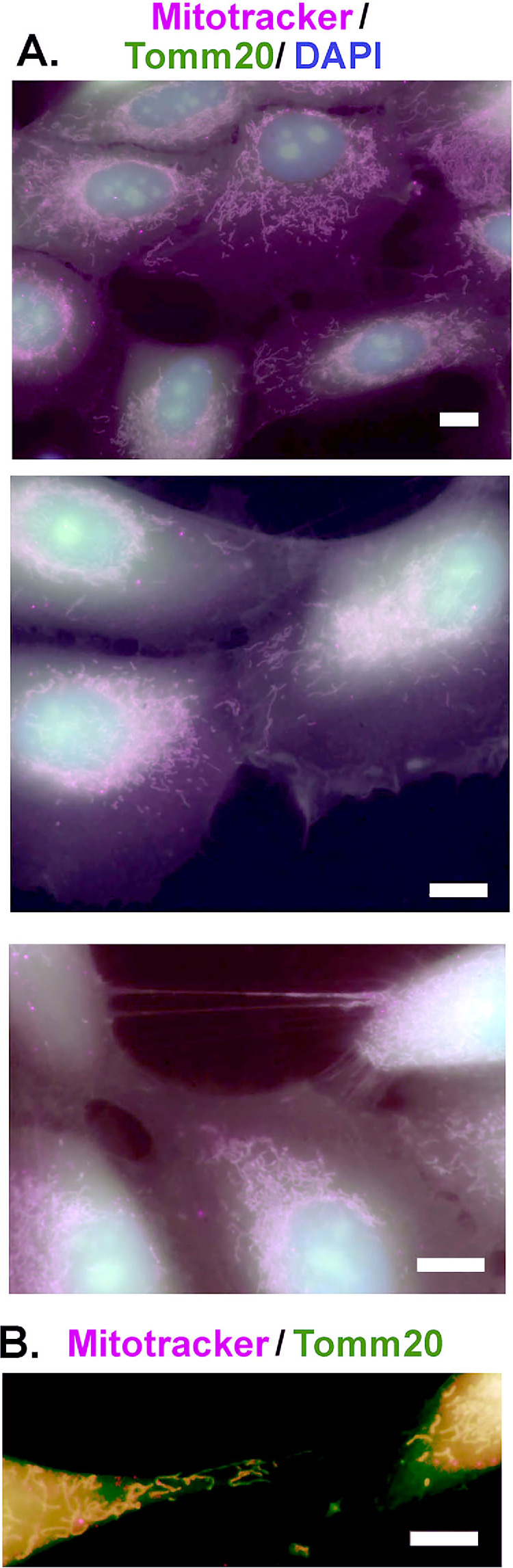
Mitotracker and Tomm20 labeling of HCLE cell mitochondria reveals that Mitotracker does not impact mitochondrial morphology and provides indirect evidence for mitochondrial transfer. HCLE cultures treated with Deep red Mitotracker, fixed, and stained with an antibody against Tomm20. Although the secondary antibody for Tomm20 shows diffuse nonspecific labeling of the HCLE cells, brightly labeled Tomm20+ mitochondria are readily observed along with mitochondria labeled with Mitotracker. (**A**) Representative images showing that Mitotracker and Tomm20 overlap and HCLE cell mitochondria are long and extended. At regions of close cell/cell contact, mitochondria can be seen extending between adjacent cells, and examples of what are referred to as tunneling nanotubes containing mitochondria are observed. (**B**) To emphasize the morphologies of the mitochondria, we masked the staining for DAPI and show two HCLE cells migrating away from one another; both leave trailing edges of their cytoplasm showing elongated mitochondria, some of which are being left behind on the substrate. These images highlight the roles played by the mitochondria and cytoskeleton in HCLE cell migration. *Scale bars*: 10 µm (**A**); 25 µm (**B**).

We have shown above that mitochondrial transfer occurs in vitro between HCLE cell donors and neuron recipients and vice versa. Axons in primary mouse trigeminal neuron cultures can extend for long distances, fuse with one another (fasciculate), and then branch off from one another (defasciculate); to reduce variability and simplify our analyses, we used HCLE cell cultures for our quantitative studies. The goals of the studies presented next are to assess the mechanisms involved in mitochondrial transfer and the roles played by cell stress, phagocytosis, gap junctions, cell adhesion, and cell migration using HCLE cells. Mitotracker Deep red is rapidly internalized into HCLE cells and their mitochondria. It is fixable and compatible with labeling cells with multiple fluorophores. To determine whether Mitotracker alters HCLE mitochondrial morphology, we next grew cells on glass coverslips coated with LN332 matrix and labeled the cells with Mitotracker; cells were then washed and fixed to allow us to colocalize Mitotracker with an antibody against Tomm20 and DAPI to show nuclei. In Figure 5A we show that the mitochondria in the HCLE cells are elongated and localized to the perinuclear region of the cells. HCLE cells in vitro grow in clusters where they associate closely with one another. At sites of close association, mitochondria appear to be transferring between cells. In one image, two structures that appear to be tunneling nanotubes are apparent between two cells, and mitochondria are present within the nanotubes. In Figure 5B, the blue DAPI color has been removed to highlight the presence of elongated mitochondria at the trailing edges of HCLE cells migrating away from one another and two mitochondria outside cells adhered to the matrix; one of the mitochondria expresses both Tomm20 and Mitotracker, but the other mitochondria express only Tomm20 consistent with mitochondrial membrane depolarization and diffusion of Mitotracker out of mitochondria before fixation. Mitochondria on substrates expressing both Tomm20 and Mitotracker would be expected to retain their functionality.

### Acid Stressing HCLE Cells Reduces Mitochondrial Content, Phagocytosis, Adhesion to LN332, and Cell Migration but Increases Mitochondrial Transfer

**Figure 6.**
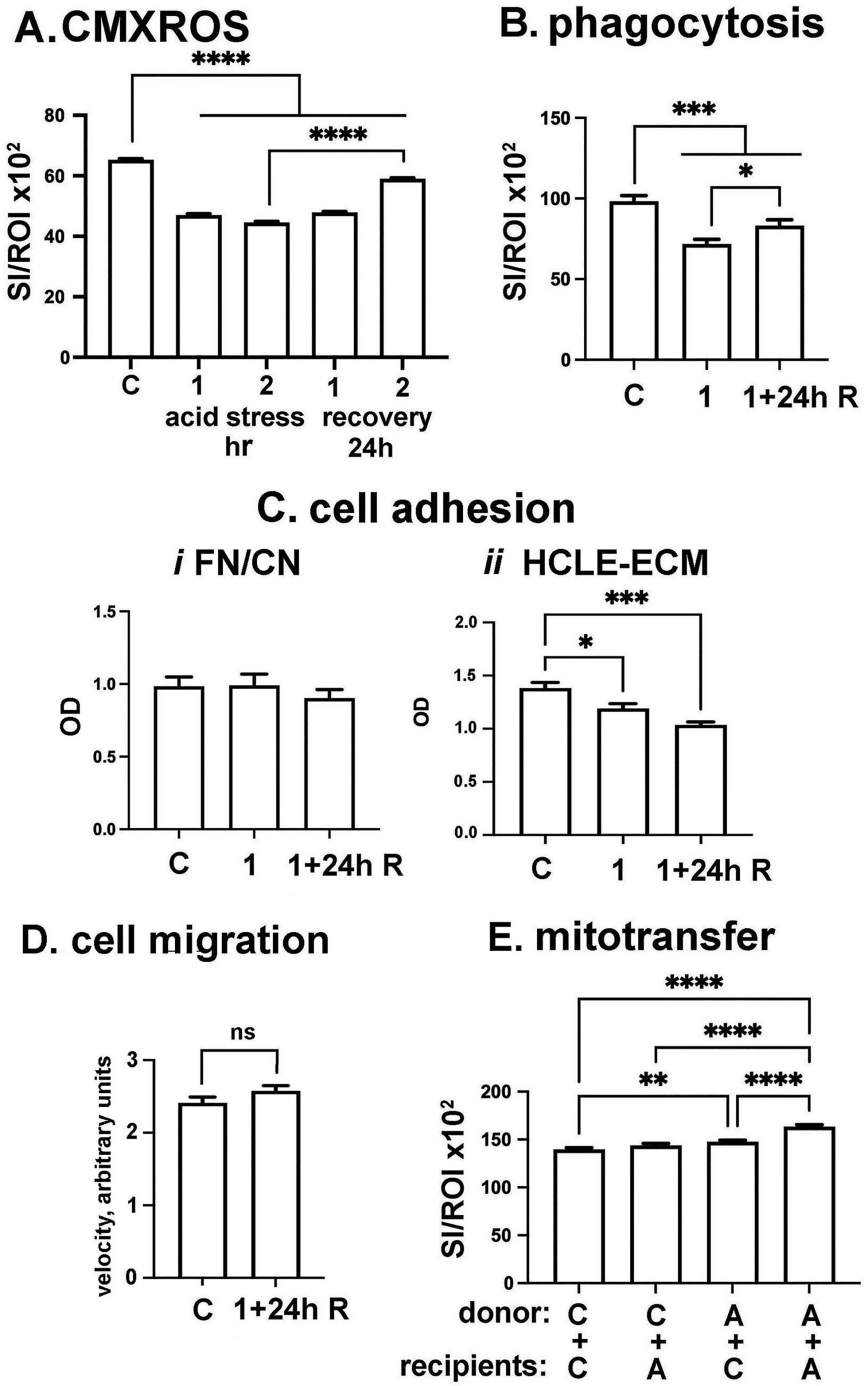
HCLE cells stressed by low pH media have fewer mitochondria and are less phagocytic but donate more mitochondria to recipient cells than control cells. (**A**) Incubating HCLE cells in acidic (pH = 6.5) media for one- and two-hour time points reduces mitochondrial content assessed using CMXROS. Cells allowed to recover in neutral pH media for 24 hours after acid treatment only partially recover mitochondrial content. (**B**) HCLE cells stressed in acidic media for one hour (*1*) are less phagocytic but partially recover phagocytic capacity when placed in neutral pH media for 24 hours before phagocytosis assay (*1+24h R*). (**C**) Cells incubated in acidic media for one hour (*1*) and allowed to recover for 24 hours in neutral pH media (*1+24h R*) adhere similarly to FN/CN as do untreated control cells; however, they show decreased adhesion to HCLE-derived ECM compared to controls**.** (**D**) HCLE cells incubated in acid media for one hour and then tracked overnight in neutral pH media (*1+24h R*) migrate with the same velocity as untreated control cells. (**E**) Control cells are referred to as donor or recipient C cells, whereas cells stressed in acidic media are referred to as donor or recipient A (acidic) cells. Acid-stressed donor HCLE cells transfer more mitochondria to acid-stressed recipient cells than to control cells. (**A**, **B**, **E**) *n* = 600 values per variable; (C) *n* = 6 values per variable assessed; and (**D**) *n* > 60 cell per variable assessed. Data were analyzed for statistical significance using Graphpad Prism. *P < 0.05; **P < 0.01; ***P < 0.001; ****P < 0.0001. No asterisks or *ns* above a graph indicates *P* ≥ 0.05.

Studies have shown that in response to transient cell stress, cells eject a portion of their mitochondria^37^ and become more likely to accept mitochondria from donor cells.^38^^,^^39^ Incubating cells transiently in media with pH 6.5, rather than the standard pH 7.0, has been shown to induce cell stress and ejection of a portion of the cells mitochondria, with recovery of mitochondrial numbers after cells are returned to normal pH media.^40^ For these studies, we lowered the pH of HCLE media to 6.5 and incubated HCLE cells in this acidic media for one and two hours and assessed mitochondrial content using CMXROS, a fluorescent molecule that enters mitochondria in cells and allows for the quantification of mitochondria. Data are presented in Figure 6A and show that incubating cells in acidic media for both one- and two-hour time points decreases mitochondrial content significantly. To determine whether cells recover mitochondrial content after acid treatment, we next incubated cells for one or two hours in acidic media, washed the cells, fed them with normal pH 7.0 media, and allowed the cells to grow for another 24 hours before assessing mitochondrial content with CMXROS. These data, also presented in Figure 6A, show that cells partially recover mitochondrial content after 24 hours in normal media. Next, we assessed the ability of cells stressed with acidic media for one hour to phagocytose opsonized beads. Acid-stressed cells were found to phagocytose 28% fewer beads than control cells as shown in Figure 6B. Allowing cells to recover in normal media for 24 hours partially reverts the reduction in cells’ ability to carry out phagocytosis. These results affirm that HCLE cells incubated in acidic media expel mitochondria and become less capable of phagocytosing particles than control cells and, although cells can at least partially recover their mitochondrial content and phagocytic capacity, recovery takes time because we found that cells do not fully recover after an overnight recovery period in normal media. These results are consistent with data on other cell types indicating that cells typically take at least 24 hours to upregulate mitochondrial biogenesis in response to cell stress.^40^

Next, we performed cell adhesion assays on HCLE cells exposed to acid media for one hour, followed by trypsinization and adhesion in normal media. Results presented in Figure 6C show that neither a one-hour incubation of HCLE cells in acid media nor allowing acid-stressed cells to recover in normal media alters HCLE adhesion to a FN/CN matrix but does reduce adhesion to the LN332-enriched matrix secreted by HCLE cells. We then asked how acid-stressing cells for one hour impacts cell migration; data are presented in Figure 6D and show that migration is not altered by low pH media. This assay quantifies cell velocity every 10 minutes over a 16-hour 40-minute time period (100 images). We then asked how acid-stressing cells impacts mitochondrial transfer. A schematic showing the workflow for mitochondrial transfer assays is presented in Figure 1. We quantify the transfer of Mitotracker-labeled mitochondria from donor cells into unlabeled recipient cells. Figure 6E shows the results obtained quantifying the transfer of mitochondria from control and acid- treated donor cells labeled with cell trace to unlabeled control and acid-treated recipient cells. After fixation, cells are stained with phalloidin, and data are expressed as the sum intensity of Mitotracker/region of interest. For each variable, 600 cells were assessed, and experiments were performed three times. Control cells are referred to as donor C or recipient C cells, whereas cells stressed in acidic media are referred to as donor A or recipient A (acidic) cells. More mitochondria from donor A cells are transferred into recipient A and recipient C cells compared to donor C cells. As shown in Figure 6A, acid-stressed cells have fewer mitochondria and are likely releasing more mitochondria into their media, whereas the reduced mitochondrial content in acid-stressed recipient cells induces cells to take in more mitochondria from donor A cells but not from donor C cells. Because phagocytosis is reduced in acid-treated cells but mitochondrial transfer is increased, these results implicate mechanisms other than phagocytosis in mediating the uptake of donated mitochondria by HCLE cells.

### Pharmacological Inhibition of Phagocytosis Increases Mitochondrial Transfer Between HCLE Cells

**Figure 7.**
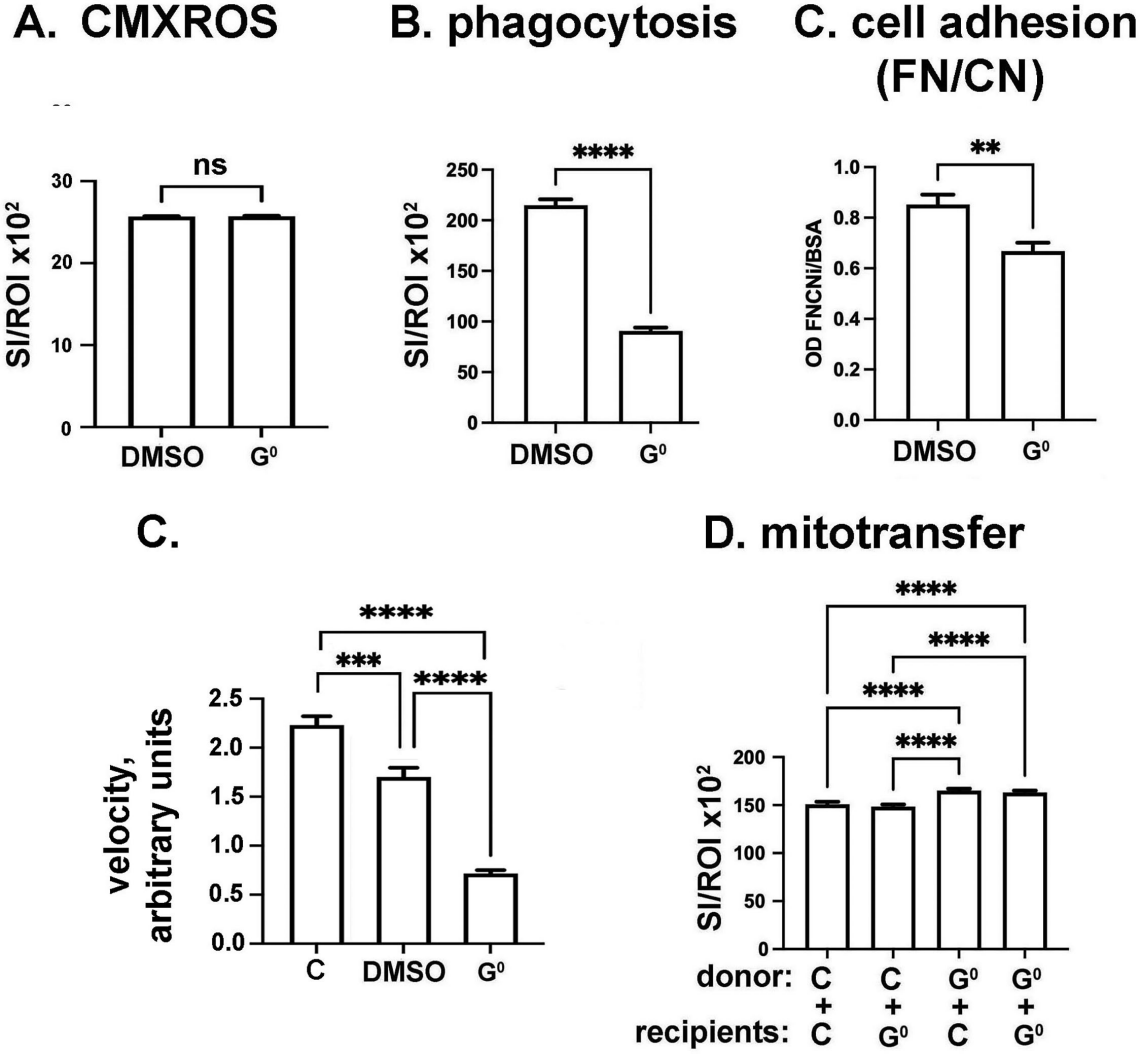
Inhibition of phagocytosis has no impact on mitochondrial content but does reduce cell adhesion and increases mitochondrial transfer. (**A**) Incubating HCLE cells in Gö6976 (*G^0^*) does not reduce mitochondrial content assessed using CMXROS. (**B**) Inhibition of phagocytosis using Gö6976 (*G^0^*) reduces phagocytosis in HCLE cells. (**C**) Pharmacological inhibition of phagocytosis using G^0^ reduces HCLE cell adhesion. (**D**) Gö6976 treatment of donor cells increase their transfer of mitochondria to both control and Gö6976-treated recipient cells. (**A**, **B**, **D**) *n* = 600 values per variable assessed; (**C**) *n* = 6 values per variable assessed. Experiments were repeated two or three times. Data were analyzed for statistical significance using Graphpad Prism. **P < 0.01; ***P < 0.001; ****P < 0.0001. *ns* above a graph indicates *P* ≥ 0.05.

To better understand the impact that phagocytosis has on mitochondrial transfer, we incubated HCLE cells with the phagocytosis inhibitor Gö6976, referred to here as G^0^. Because G^0^ is prepared using DMSO, vehicle and G^0^-treated cells were incubated in media containing the same concentration of DMSO vehicle. In Figure 7A we show that, G^0^ does not reduce mitochondrial content assessed by CMXROS. In Figure 7B we show that, as expected, G^0^ reduces HCLE phagocytosis; in Figure 7C we show that G^0^ also reduces cell adhesion. For mitochondrial transfer assays, experiments were performed using vehicle-treated control donors and recipients or G^0^-treated donors and recipients. Results are presented in Figure 7D and indicate that G^0^ treatment significantly increases mitochondrial transfer from donor cells to control and G^0^ -treated HCLE recipients. Recipient cell G^0^ treatment has no impact on mitochondrial transfer. These results, along with those presented for acid-stressed cells above, indicate that the ability of cells to engage in phagocytosis does not correlate with a cell’s ability to internalize mitochondria donated by other cells.

### Pharmacological Inhibition With the Gap Junction Inhibitor 18α-Glycyrrhetinic Acid Reduces Phagocytosis, Cell Adhesion, Cell Migration, and Mitochondrial Transfer in HCLE Cells

**Figure 8.**
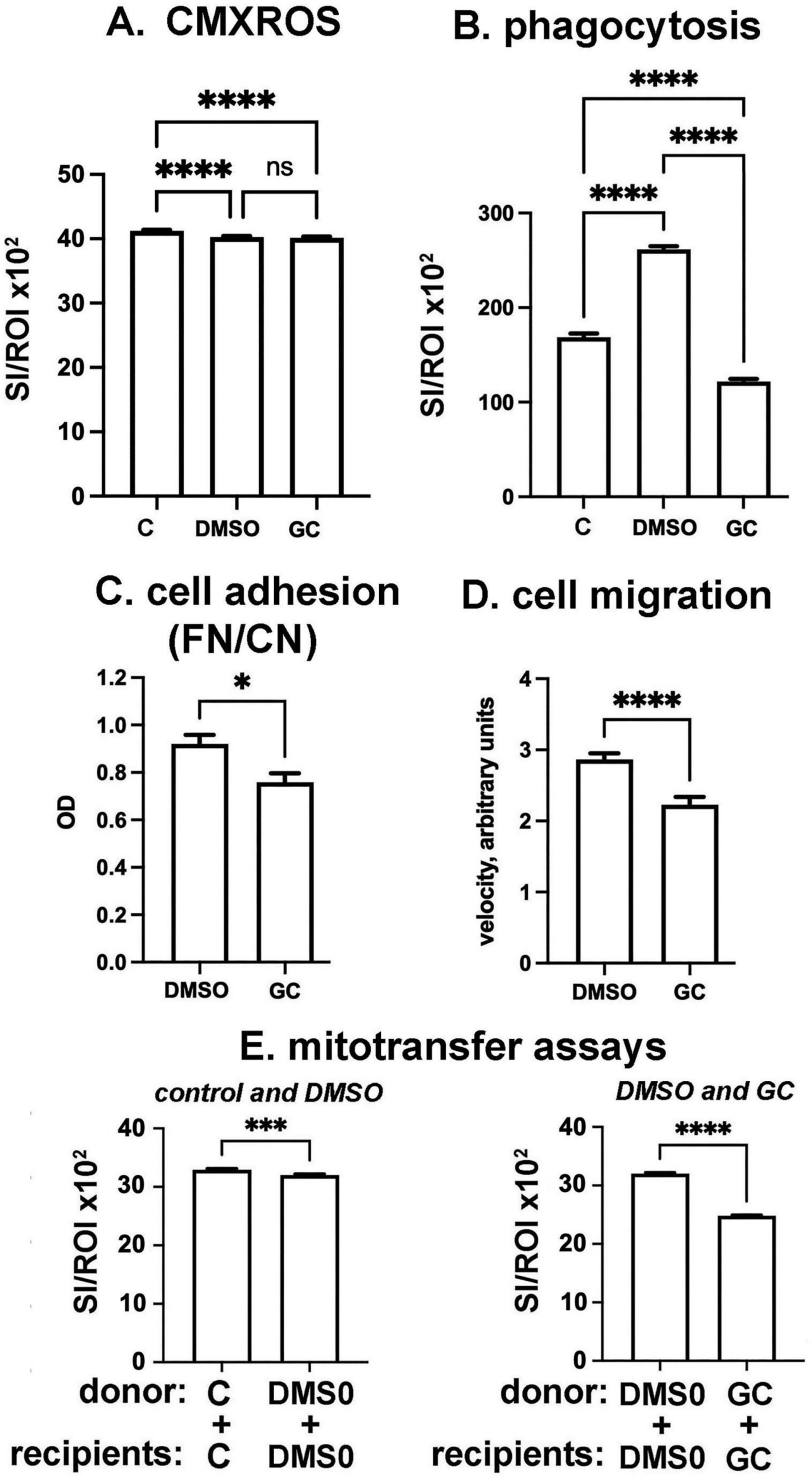
Inhibiting gap junctions with 18α-glycyrrhetinic acid (*GC*) reduces phagocytosis, cell adhesion, migration, and mitochondrial transfer between HCLE cells but does not impact mitochondrial content. (**A**) Although treating HCLE cells with DMSO vehicle reduces CMXROS, there is no difference in CMXROS when GC-treated cells are compared to DMSO vehicle–treated cells. (**B**) DMSO, used as a vehicle at the same concentrations as GC, increases phagocytosis compared to control cells, but GC reduces phagocytosis compared to DMSO vehicle. (**C**) GC reduces cell adhesion compared to vehicle alone. (**D**) GC reduces cell migration compared to vehicle alone. (**E**) DMSO in vehicle increases mitochondrial transfer between HCLE cells slightly. However, GC reduces mitochondrial transfer significantly between HCLE cells compared to vehicle-treated controls. (**A**, **B**, **E**) *n* = 600 for each variable assessed; (**C**) *n* = 6 for each variable assessed; (**D**) *n* > 60 for each variable assessed. Data were analyzed for statistical significance using Graphpad Prism. *P < 0.05; ***P < 0.001; ****P < 0.0001. No asterisks or *ns* above a graph indicates *P* ≥ 0.05.

Studies on other cell types have shown a role for gap junctions in mitochondrial transfer, but none have been performed on corneal epithelial cells. Data suggest that functional gap junctions play roles in mediating the adhesion between donor and recipient cell membranes to permit mitochondria to move between cells.^7^ To better understand how gap junctions are involved in mitochondrial transfer we used the well characterized gap junction inhibitor 18α-glycyrrhetinic acid (GC) in HCLE CMXROS, phagocytosis, cell adhesion, cell migration, and mitochondrial transfer assays. Because GC stock solutions are prepared using DMSO as a solvent, vehicle-treated cells were incubated in the same concentration of DMSO as used for GC; the concentration of DMSO in media did not exceed 0.02%. In Figure 8A, data for mitochondrial content assessed using CMXROS are presented. Data show that although the DMSO vehicle reduces CMXROS, GC treatment does not alter CMXROS when compared to the vehicle control. In Figure 8B, data for phagocytosis by HCLE cells are presented. DMSO vehicle in media has a significant impact on phagocytosis, increasing it by 30% to 40% compared to controls cells. GC decreases phagocytosis compared to DMSO vehicle. Data for cell adhesion are presented in Figure 8C. GC inhibits HCLE adhesion compared to vehicle. Data for cell migration are presented in Figure 8D and show that GC treatment slows HCLE cell migration. These data indicate important roles for gap junctions in phagocytosis, adhesion, and cell migration in HCLE cells. Mitochondrial transfer assays are presented in Figure 8E and show that DMSO vehicle alone reduces mitochondrial transfer between cells by 3% whereas GC reduces mitochondrial transfer significantly by 22%. Taken together, the studies presented show that mitochondrial transfer rates are positively impacted by acid stress and inhibition of phagocytosis, whereas interfering with gap junctions impairs mitochondrial transfer despite reducing phagocytosis, adhesion, and migration.

### Treating HCLE Cells With RI Reduces Mitochondrial Content, Phagocytosis, Cell Migration, and Matrix Deposition but Increases Cell Adhesion and Mitochondrial Transfer

**Figure 9.**
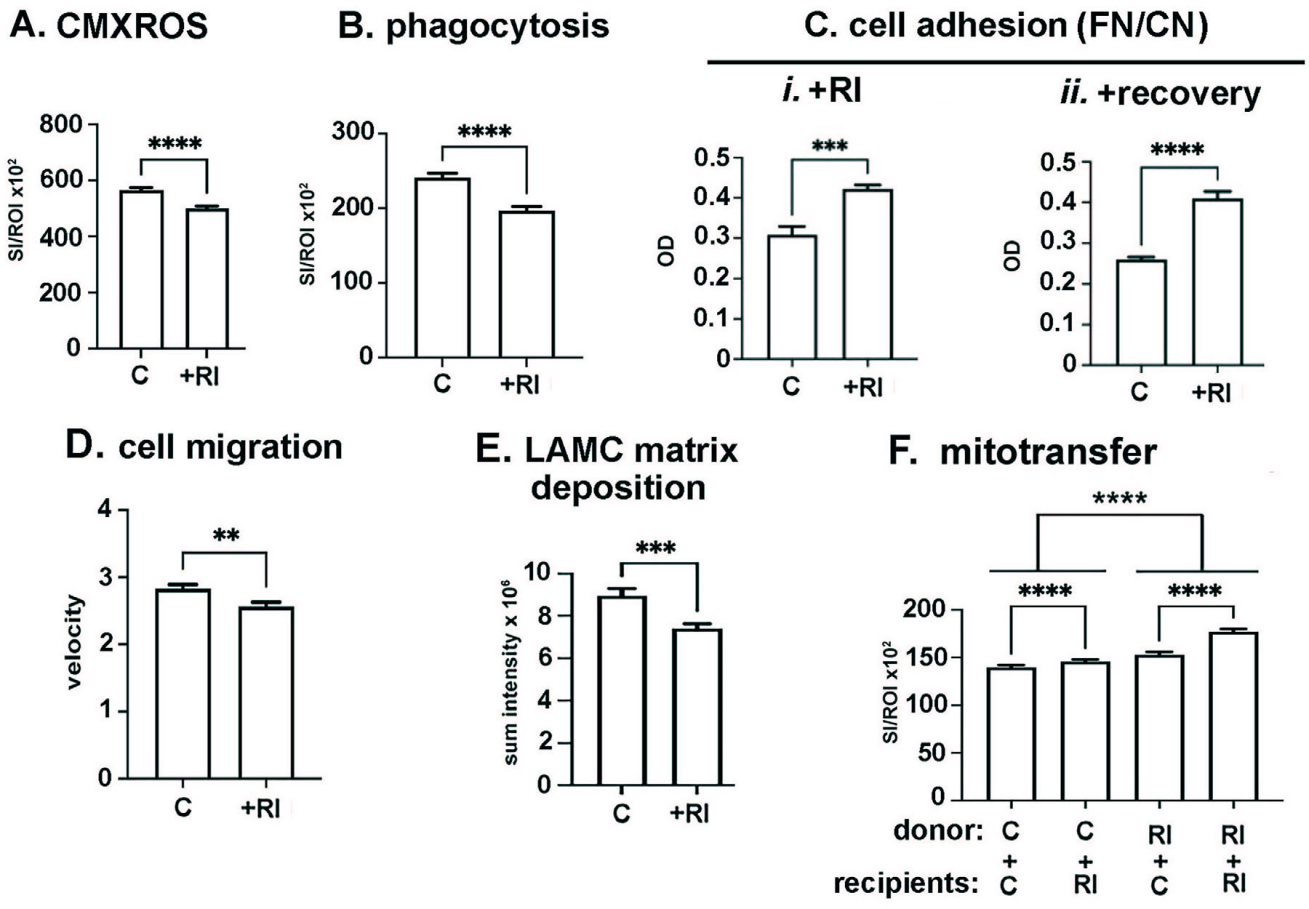
RI treatment decreases mitochondria content, phagocytosis, and cell migration but increases HCLE adhesion and mitochondrial transfer. Cells grown in HCLE media with or without 5 µM RI overnight before these assays were performed. (**A**) RI treatment decreases mitochondrial content. (**B**) RI treatment decreases phagocytosis. (**C*i***) RI treatment increases HCLE adhesion to FN/CN**. (C*ii***) The RI-induced increase in cell adhesion to FN/CN is maintained in cells allowed to recover in media without RI for 24 hours. (**D**) RI treatment reduces HCLE migration rates. (**E**) The ability of HCLE cells to secrete and assemble an insoluble LAMC extracellular matrix is reduced. (**F**) RI treatment of both HCLE donors and recipients increases mitochondrial transfer. (**A**, **B**, **F**) *n* = 600 for each variable assessed; (**C**) *n* = 6 for each variable assessed; (**D**) *n* > 60 for each variable assessed; (**E**) *n* = 6 for each variable assessed. Data were analyzed for statistical significance using Graphpad Prism. **P < 0.01; ***P < 0.001; ****P < 0.0001.

We next assessed the impact of overnight incubation of cells with RI. In Figure 9A we show that RI treatment reduces mitochondrial content significantly by 10%, and in Figure 9B we show that RI treatment reduces HCLE phagocytosis significantly by 26%. Rho kinases can also impact cell adhesion; next we assessed the impact of RI treatment on HCLE adhesion. In Figures 9C*i* and 9C*ii* we demonstrate that RI-treated HCLE cells are more adhesive than control cells, and this difference persists for at least 24 hours after RI is removed from media, cells washed, and allowed to recover in media without RI. In In Figure 9D we quantify cell migration in RI treated cells and show that RI cells migrate slower than control cells. Increased ROCK activity has been shown to lead to pulmonary fibrosis^41^ and RIs have been used to reduce matrix deposition in fibroblasts.^42^ We next looked at whether RI- treated HCLE cells deposit the same amount of LAMC-containing matrix as control cells. As shown in Figure 9E, RI treatment reduces LAMC matrix deposition by HCLE cells.

In Figure 9F we present data showing mitochondrial transfer between control and RI-treated donor cells and control and RI-treated recipients. RI treatment significantly enhances mitochondrial transfer of donated mitochondria regardless of whether donor or recipient cells are exposed to RI. The largest change in mitochondrial transfer is observed when control donors and recipients are compared to RI-treated donors and recipients: Mitochondrial transfer increases by 20% compared to controls when donor and recipient cells are both treated with RI.

### Media Conditioned by HCLE Cells Previously Exposed to RI (CMRI) Also Reduces Mitochondrial Content and Matrix Deposition and Increases Cell Adhesion and Mitochondrial Transfer, but Its Impact Is Less Than That Observed for Direct RI Treatment

**Figure 10.**
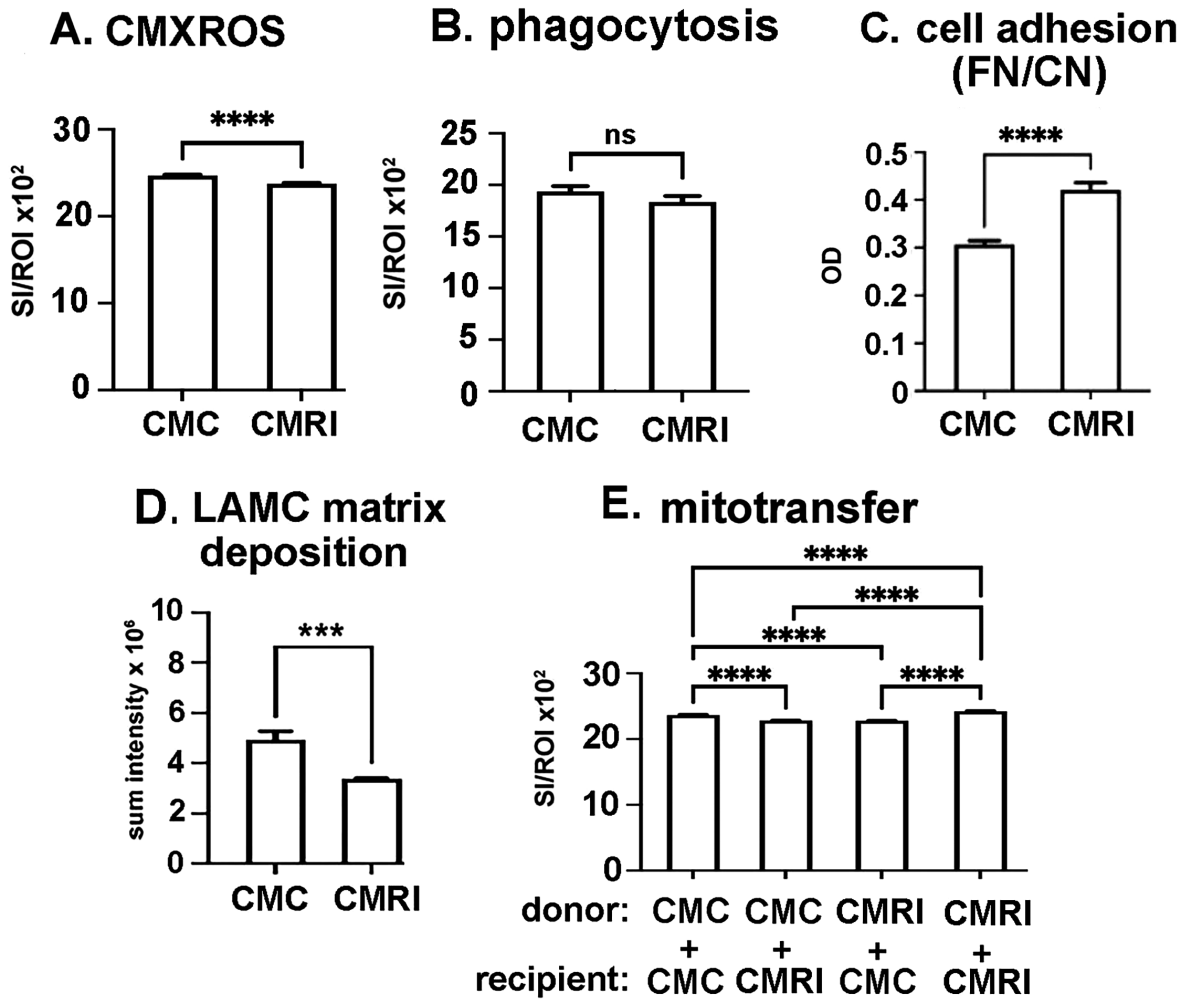
Media conditioned by HCLE cells previously exposed to ROCK inhibitor also reduces mitochondrial content and matrix deposition and increases cell adhesion and mitochondrial transfer. (**A**) Cells grown in CMRI have reduced mitochondrial content compared to cells grown in CMC. (**B**) Cells grown in CMRI phagocytose similar numbers of beads as cells grown in CMC. (**C**) Cells grown in CMRI are more adherent than cells grown in CMC. (**D**) Cells grown in CMRI reduced amounts of insoluble LAMC extracellular matrix than cells grown in CMC. (**E**) Mitochondrial transfer is slightly but significantly greater in cells grown in CMRI compared to cells grown in CMC. (**A**, **B**, **E**) *n* = 600 for each variable assessed; (**C**, **D**) *n* = 6 for each variable assessed. Data were analyzed for statistical significance using Graphpad Prism. ***P < 0.001; ****P < 0.0001. *ns* above a graph indicates *P* ≥ 0.05 and data that were not statistically significant.

We have shown previously that media conditioned by transiently MMC-treated HCLE cells can impact cell adhesion and migration by altering cytokine secretion.^27^ To determine whether the changes induced by RI treatment cause HCLE cells to secrete proteins and other factors into media that can impact mitochondria transfer, conditioned media was prepared from control (CMC) and RI-treated cells (CMRI) (see methods section and Supplementary Figure S1 for CM preparation). HCLE cells were grown overnight in CMC or CMRI. In Figure 10A we show that although CMRI reduces HCLE mitochondrial content significantly, the reduction is less than 4% compared to the 10% reduction caused by treating cells directly with RI (see Fig. 9A). We next assessed the impact of CMRI on phagocytosis; Figure 10B shows that there was no significant difference in phagocytosis observed for HCLE cells growing in CMC and CMRI; however, in Figure 9B we show a significant decrease in phagocytosis in cells treated directly with RI. We next assessed the impact of CMC and CMRI on HCLE adhesion. In Figure 10C we show that cells growing in CMRI are more adhesive, and in Figure 10D we show that cells growing in CMRI secrete less matrix than cells growing in CMC; the differences in adhesion and matrix deposition are similar to those seen when cells are directly treated with RI. Data on the impact of CMC and CMRI on mitochondrial transfer are presented in Figure 10E. Although mitochondrial transfer is significantly higher for CMRI donors and CMRI recipients compared to CMC donors and CMC recipients, the difference is just 2% compared to the 20% seen for mitochondrial transfer for direct RI-treated cells to control cells (see Fig. 9F).

### Exposing HCLE: Trigeminal Neuron Co-Cultures to Neurobasal Media Conditioned by RI-Treated HCLE Cells Enhances Mitochondrial Extrusion by HCLE Cells

**Figure 11.**
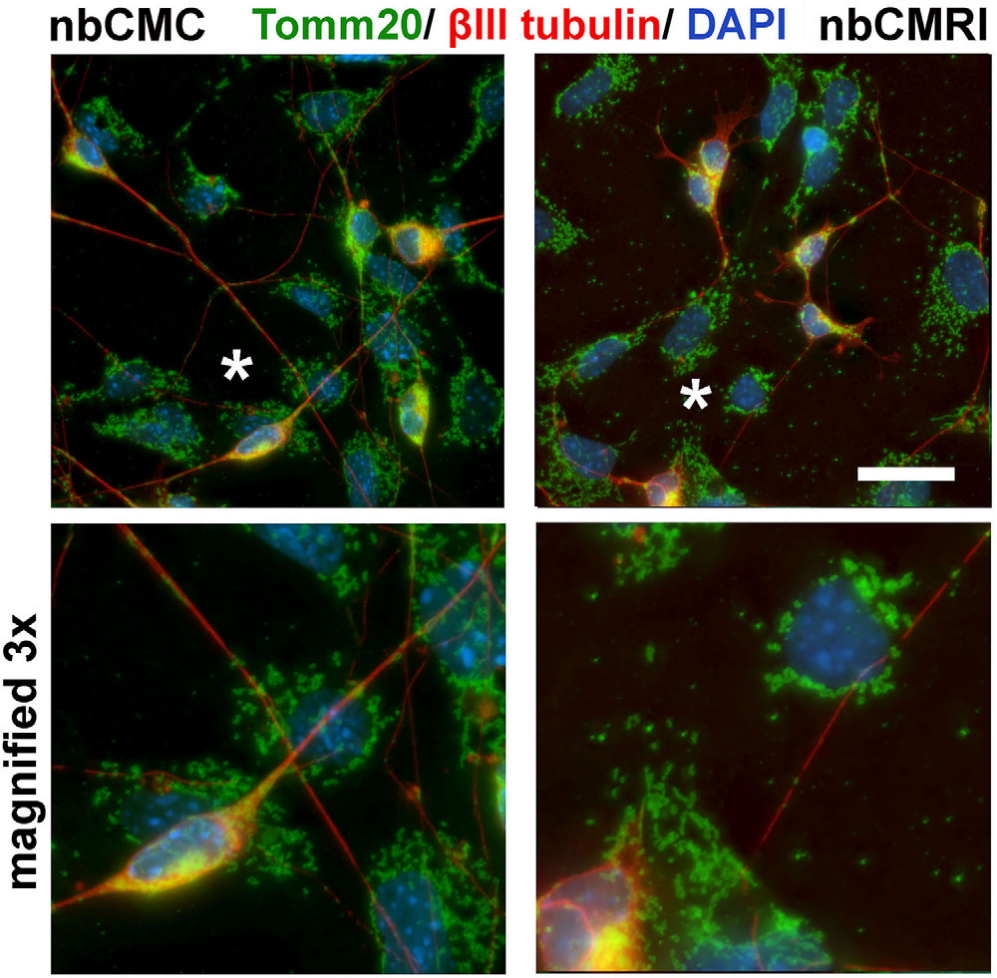
Co-cultures growing in conditioned media from RI treated cells release mitochondria. Co-cultures of HCLE cells and primary TG neurons were grown in Neurobasal conditioned medium from control HCLE cells (*nbCMC*) or from HCLE cells that had been previously treated with RI (*nbCMRI*). After fixation, cultures were stained to reveal mitochondria using a Tomm20 antibody (*green*) and axons using a βIII tubulin antibody (*red*) with nuclei stained with DAPI (*blue*). Numerous Tomm20+ mitochondria can be visualized in axons and HCLE cells in both CMC and CMRI cultures, but CMRI cultures show mitochondria that have been released from cells and adhere to glass coverslips. *Scale bar:* 10 µm; sites in images on the top highlighted with *asterisks* were magnified ×3 digitally and are presented at the bottom.

To determine whether exposure of HCLE cells to conditioned media from RI-treated cells impacts mitochondrial morphology and localization in HCLE-neuron co-cultures, we generated HCLE-neuron co-cultures and grew them in defined nb-conditioned media (CM) from control cells (nbCMC) and cells treated with RI (nbCMRI) overnight. The HCLE cells and neurons in the nbCMRI co-cultures had not been exposed directly to RI but to molecules secreted by HCLE cells exposed to RI. After fixation, cultures were stained with an antibody to Tomm20 (green) to reveal mitochondria and βIII tubulin (red) to reveal neurons and their axons and data presented in Figure 11. Tomm20 staining in the co-cultures grown in nbCMC is restricted to HCLE cells, neuron cell bodies and within axons and mitochondria; by contrast, Tomm20 staining in the co-cultures grown in nbCMRI show numerous Tomm20+ mitochondria adhered to the cell culture substrates between HCLE cells and axons. Although both HCLE cells and neurons contain mitochondria, because the numbers of neurons in these co-cultures is low, it is likely that the mitochondria that adhere to the tissue culture substrate were released by HCLE cells.

## Discussion

We set out here to determine how mitochondrial transfer is regulated in corneal epithelial cells. Currently, the most likely mechanisms that regulate the transfer of mitochondria into cells are considered to be tunneling nanotubes and internalization of free mitochondria or mitochondria within EVs.^4^^,^^6^ Gap junctions are implicated in facilitating mitochondrial transfer via unknown mechanisms.^4^ Although internalization of EVs and free mitochondria has been assumed to take place via phagocytosis, differentiating between phagocytosis and macropinocytosis is challenging.^43^ Different cell types regulate these processes differently, and both involve many of the same proteins. The data presented here affirm important roles for gap junctions but shift attention away from phagocytosis as the primary mechanism mediating mitochondrial internalization by corneal epithelial cells.

**Figure 12.**
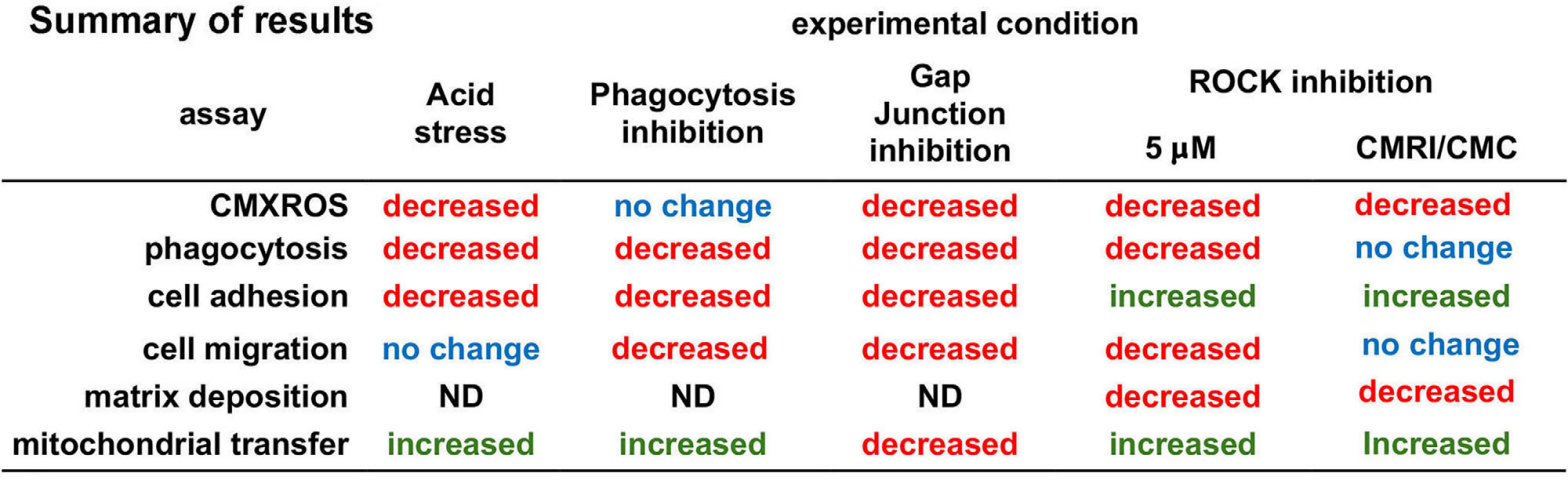
Summary results from procedures performed. Text in *red* and *green* indicate data, presented in Figures 6–10, that are either decreased or increased in response to the experimental condition indicated. *Blue* text indicates data that are not significantly different, and ND (not determined) indicates data that have not been obtained.

Figure 12 summarizes the data presented in Figures 6 to 10. Although tunneling nanotubes may well mediate mitochondrial transfer in vitro in the corneal epithelium, nanotubes are less likely to play major roles in vivo because of the close approximation of cell membranes to one another, as well as the high density of desmosomes, adherens junctions, and gap junctions in the cell membranes of the epithelial cells. Given our studies showing that corneal epithelial cells ingest axonal debris after trephine injury,^3^ probing into the role played by phagocytosis was essential. However, we find that inhibiting phagocytosis directly using the compound Gö6976 (G^0^) does not decrease mitochondrial transfer. Instead, it increases the ability of HCLE cells to donate mitochondria to recipient cells but does not alter the ability of recipient cells to internalize donated mitochondria. Two other stresses (acid stress and RI treatment) decreased HCLE phagocytosis but increase mitochondrial transfer. Only inhibition with the gap junction inhibitor GC reduces phagocytosis and also reduces mitochondrial transfer.

When we inhibit gap junctions in our HCLE cells using GC, we also reduce mitochondrial content, phagocytosis, cell adhesion, cell migration, and mitochondrial transfer. These data confirm for the first time that gap junctions in vitro play a role in the ability of corneal epithelial cells to phagocytose debris and donate and receive mitochondria from other cells. In mammals, gap junction hemichannels are composed of heximers of connexin family member proteins; all six connexins can either be the same (homoheximer), or they can comprise several different connexin family members (heteroheximer). The canonical function of gap junctions requires two hemichannels on adjacent cells to form a channel that allows molecules under 5 nm in diameter to pass between the cells. Recently, gap junction proteins have been shown to have a number of non-canonical functions and play roles in facilitating cell adhesion and enhancing the uptake of EVs.^44^^,^^45^

A study published previously showed that RI enhances mitochondrial transfer between corneal epithelial cells^15^; here we confirm and extend those data to show that 5 µM RI treatment also reduces mitochondrial content while inhibiting phagocytosis, cell adhesion, and cell migration. Stressing cells by treating them with acid media for one to two hours also reduces mitochondrial content and increases mitochondrial transfer. Taken together, our results indicate a role for cell stress in enhancing mitochondrial transfer by HCLE cells and confirm that mitochondrial transfer is regulated by mechanisms distinct from those that regulate phagocytosis.

How mitochondrial transfer takes place in vivo, in the cornea, in response to injury is under intense study. Our recent in vivo study in mice showed that topical RI treatment at the time of injury increases axonal regeneration after trephine injury in female but not male mice.^46^ The trephine injury model severs 40% to 50% of the intraepithelial corneal nerves and induces corneal epithelial cells to ingest axonal debris and mitochondria from the severed axons.^3^ Axonal debris can be seen accumulating within LAMP1-positive lysosomes in the corneal epithelial basal and suprabasal cells within six hours of injury.^1^ However, corneal epithelial cells are able to support complete reinnervation of the sensory axons within several days of injury.

In the cytoplasm of control corneal epithelial basal cells, FIBSEM studies show abundant glycogen granules^3^; within five to six hours after trephine injury, those granules had been metabolized, indicating a rapid increase in corneal epithelial cell metabolism in response to trephine injury. The transfer of mitochondria from corneal sensory nerves to epithelial cells would provide energy needed to support efficient removal of axonal debris.

Studies of how cells ingest nutrients and debris have identified specific and nonspecific endocytic pathways used by cells during homeostasis and in response to injury and stress. Specific pathways include receptor-mediated endocytosis, and nonspecific pathways include micropinocytosis and macropinocytosis.^43^^,^^47^ In macropinocytosis, cells ingest fluid from their environment that can contain particles such as debris and mitochondria via non-receptor-mediated mechanisms by extending membrane extrusions referred to as ruffles or lamellipodia that collapse. The tips of the ruffles fuse with their cell membrane, trapping extracellular fluid and particles in vacuoles referred to as macropinosomes.^48^ Macropinosomes can be recycled back to the plasma membrane or undergo maturation within the cells endocytic compartment.^47^ Macropinocytosis has been reported to mediate the internalization of isolated functional mitochondria by mesenchymal cells^49^; our results suggest that macropinocytosis mediates mitochondrial transfer in corneal epithelial cells, but further research is needed. Controversy exists regarding the specificity of the macropinocytosis inhibitors characterized thus far. Amiloride blocks macropinocytosis in macrophages but not in dendritic cells. Other inhibitors block both phagocytosis and macropinocytosis in some cell types but not others.^50^ RIs have been reported to promote membrane ruffling in fibroblasts^51^ and cancer cells,^52^ which would enhance the formation of macropinosomes.

Here we show that both direct (RI) and indirect treatment (CMRI) of HCLE cells in vitro increase HCLE uptake of mitochondria. These results, coupled with in vivo results showing enhanced reinnervation after trephine injury after RI treatment,^46^ indicate important roles for mitochondrial transfer in mediating the ability of corneal epithelial cells to support the intraepithelial corneal nerves during homeostasis and in response to injury. Further studies of the potential roles played by ROCK inhibition, gap junctions, and macropinocytosis in mitochondrial transfer in corneal epithelial cells and in the sensory nerves that innervate them are needed.

## Supplementary Material

Supplement 1
