# Supplementary material for: Mechanisms Regulating Mitochondrial Transfer in Human Corneal Epithelial Cells
Source: Invest Ophthalmol Vis Sci. 2024 Nov 6;65(13):10. doi: 10.1167/iovs.65.13.10 (PMC11549930; doi:10.1167/iovs.65.13.10)

Supplemental Figure 1: Preparation of Conditioned Media. Schematic showing the workflow used for preparation of conditioned media

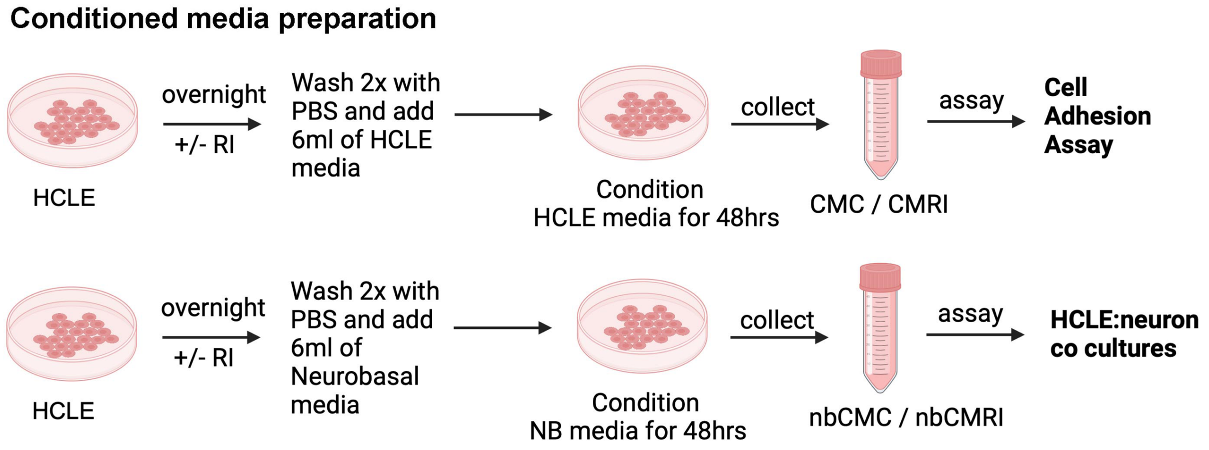

Supplement: Supplement 1 [file iovs-65-13-10_s001.pdf]
